# Towards optimal treatment selection for borderline personality disorder patients (BOOTS): a study protocol for a multicenter randomized clinical trial comparing schema therapy and dialectical behavior therapy

**DOI:** 10.1186/s12888-021-03670-9

**Published:** 2022-02-05

**Authors:** Carlijn J. M. Wibbelink, Arnoud Arntz, Raoul P. P. P. Grasman, Roland Sinnaeve, Michiel Boog, Odile M. C. Bremer, Eliane C. P. Dek, Sevinç Göral Alkan, Chrissy James, Annemieke M. Koppeschaar, Linda Kramer, Maria Ploegmakers, Arita Schaling, Faye I. Smits, Jan H. Kamphuis

**Affiliations:** 1 Department of Clinical Psychology, University of Amsterdam, Nieuwe Achtergracht 129-B, Amsterdam, 1018 WS the Netherlands; 2 Department of Neurosciences, Mind Body Research, KU Leuven, Herestraat 49, 3000 Leuven, Belgium; 3 Department of Addiction and Personality, Antes Mental Health Care, Max Euwelaan 1, Rotterdam, 3062 MA the Netherlands; 4 Institute of Psychology, Erasmus University Rotterdam, P.O. Box 1738, Rotterdam, 3000 DR the Netherlands; 5 Arkin Mental Health, NPI Institute for Personality Disorders, Domselaerstraat 128, Amsterdam, 1093 MB the Netherlands; 6 PsyQ Personality Disorders Rotterdam-Kralingen, Max Euwelaan 70, Rotterdam, 3062 MA the Netherlands; 7 i-psy Amsterdam, Overschiestraat 61, Amsterdam, 1062 XD the Netherlands; 8 Department of Personality Disorders, Outpatient Clinic De Nieuwe Valerius, GGZ inGeest, Amstelveenseweg 589, Amsterdam, 1082 JC the Netherlands; 9 PsyQ Amsterdam, Overschiestraat 57, Amsterdam, 1062 XD the Netherlands; 10 GGZ Noord-Holland-Noord, Stationsplein 138, 1703 WC Heerhugowaard, the Netherlands; 11 Pro Persona, Siependaallaan 3, Tiel, 4003 LE the Netherlands; 12 Pro Persona, Willy Brandtlaan 20, Ede, 6716 RR the Netherlands; 13 GGZ Rivierduinen, Sandifortdreef 19, Leiden, 2333 ZZ the Netherlands

**Keywords:** Borderline personality disorder, Schema therapy, Dialectical behavior therapy, Randomized clinical trial, Treatment selection, Personalized medicine, Mechanisms of change, Mediators, Effectiveness

## Abstract

**Background:**

Specialized evidence-based treatments have been developed and evaluated for borderline personality disorder (BPD), including Dialectical Behavior Therapy (DBT) and Schema Therapy (ST). Individual differences in treatment response to both ST and DBT have been observed across studies, but the factors driving these differences are largely unknown. Understanding which treatment works best for whom and why remain central issues in psychotherapy research. The aim of the present study is to improve treatment response of DBT and ST for BPD patients by a) identifying patient characteristics that predict (differential) treatment response (i.e., treatment selection) and b) understanding how both treatments lead to change (i.e., mechanisms of change). Moreover, the clinical effectiveness and cost-effectiveness of DBT and ST will be evaluated.

**Methods:**

The BOOTS trial is a multicenter randomized clinical trial conducted in a routine clinical setting in several outpatient clinics in the Netherlands. We aim to recruit 200 participants, to be randomized to DBT or ST. Patients receive a combined program of individual and group sessions for a maximum duration of 25 months. Data are collected at baseline until three-year follow-up. Candidate predictors of (differential) treatment response have been selected based on the literature, a patient representative of the Borderline Foundation of the Netherlands, and semi-structured interviews among 18 expert clinicians. In addition, BPD-treatment-specific (ST: beliefs and schema modes; DBT: emotion regulation and skills use), BPD-treatment-generic (therapeutic environment characterized by genuineness, safety, and equality), and non-specific (attachment and therapeutic alliance) mechanisms of change are assessed. The primary outcome measure is change in BPD manifestations. Secondary outcome measures include functioning, additional self-reported symptoms, and well-being.

**Discussion:**

The current study contributes to the optimization of treatments for BPD patients by extending our knowledge on “Which treatment – DBT or ST – works the best for which BPD patient, and why?”, which is likely to yield important benefits for both BPD patients (e.g., prevention of overtreatment and potential harm of treatments) and society (e.g., increased economic productivity of patients and efficient use of treatments).

**Trial registration:**

Netherlands Trial Register, NL7699, registered 25/04/2019 - retrospectively registered.

**Supplementary Information:**

The online version contains supplementary material available at 10.1186/s12888-021-03670-9.

## Background

Borderline personality disorder (BPD) is a complex and severe mental disorder, characterized by a pervasive pattern of instability in emotion regulation, self-image, interpersonal relationships, and impulse control [1, 2]. The prevalence in the general population is estimated to be between 1 and 3% [3–5], and 10 to 25% among psychiatric outpatient and inpatient individuals [3]. BPD is associated with severe functional impairment, high rates of comorbid mental disorders, and physical health problems [5–7]. In addition, BPD is characterized by low quality of life; lower compared to other common mental disorders such as depressive disorder, and comparable to that of patients with severe physical conditions, such as Parkinson’s disease and stroke [8]. Moreover, BPD is related to a high risk of suicide (3–6%, or even up to 10% [9, 10]) and suicide attempts or threats (up to 84% [11, 12]), and an increased mortality rate [13]. Besides the detrimental effects of BPD on the individual patient, BPD also poses a high financial burden to society. BPD patients make extensive use of treatment services resulting in markedly higher healthcare costs of people with BPD compared to people with other mental disorders, such as other personality disorders [14] and depressive disorder [15]. BPD is also associated with high non-healthcare costs, including costs related to productivity losses, informal care, and out-of-pocket costs [16, 17].

### Interventions: dialectical behavior therapy and schema therapy

BPD has traditionally been viewed as one of the most difficult mental disorders to treat [18]. During recent years, a number of promising treatments have been developed and evaluated, including Dialectical Behavior Therapy (DBT) [19, 20] and Schema Therapy (ST) [21, 22]. DBT is a comprehensive cognitive behavioral treatment for BPD, rooted in behaviorism, Zen and dialectical philosophy [19]. ST is based on an integrative cognitive therapy, combining cognitive behavior and experiential therapy techniques with concepts derived from developmental theories, including attachment theory, and psychodynamic concepts [23]. For detailed information about these treatments, the reader is referred to the Methods/design section.

Several studies have demonstrated the effectiveness and the efficacy of DBT and ST for BPD, although the evidence is mostly based on low-to-moderate-quality evidence, and trials focusing on DBT, but especially ST, are limited [24, 25]. In addition, substantial reductions in direct and indirect healthcare costs have been found for both treatments [26]. However, research on the comparative effectiveness and cost-effectiveness of the two interventions is lacking. Moreover, research on mediators and moderators of treatment effects is limited. This gap warrants attention, as treatment effectiveness can be optimized by identifying mechanisms within treatments that are associated with improvement and patient characteristics that predict (differential) treatment response [27]. Optimizing treatment effectiveness of DBT and ST for BPD is highly needed since a substantial proportion of patients does not respond fully to either DBT or ST. A systematic review found a mean percentage of non-response of 46% among BPD patients treated with specialized psychotherapies, including DBT and ST [28]. In addition, more than one-third of the patients did not achieve a reliable change in BPD symptoms or even showed an increase in BPD severity after DBT or ST [29–31]). Finally, dropout rates up to 30% have been found for DBT and ST [32, 33]. Individual differences in responses to both ST and DBT have been observed across studies, but the factors driving these differences in treatment response among BPD patients are largely unknown. This state of affairs leaves the principal question “What treatment, by whom, is most effective for this individual with that specific problem, under which set of circumstances?” ([34], p111), historically one of the key questions dominating the psychotherapy research agenda, fully open in the treatment of BPD individuals [35, 36]. Identifying factors that specify which patients will benefit most from which treatment (i.e., treatment selection, or also known as precision medicine or personalized medicine; [37, 38]) will lead to fewer mismatches between patients and treatments, and in turn to better outcome and more efficient use of healthcare resources.

### Treatment selection

Several factors predicting treatment response irrespective of type of treatment (i.e., prognostic factors; [35]) among BPD patients have been reported in the literature. The overwhelming list of candidate variables and the general lack of replication hampers the research among BPD patients on prognostic factors [39]. Research among BPD patients on prescriptive factors (i.e., factors that predict different outcomes depending on the treatment; moderators) is very scarce indeed. Arntz et al. [39] examined the effect of several potential predictors of (differential) treatment response across ST and Transference Focused Psychotherapy (TFP) among BPD patients. The authors failed to find prescriptive factors, but it should be noted that the sample size was inadequate to detect subtle differences between treatments. In addition, Verheul et al. [40] found that patients with a high frequency of self-mutilating behavior before treatment were more likely to benefit from DBT compared to treatment as usual, whereas for patients with a low frequency of self-mutilating behavior effectiveness did not differ.

Historically, research has focused on a single variable to predict treatment response, but often failed to find consistent and clinically meaningful moderators [41–44]. However, it is highly unlikely that a single variable is responsible for the differences in treatment response [43, 45, 46]. In recent decades, novel approaches combining multiple predictors to determine the optimal treatment for a particular patient have been introduced, including the methods of Kraemer ([47]; optimal composite moderator) and DeRubeis and colleagues ([35]; statistically derived selection algorithm). Several studies have found that a combination of predictors was predictive of differential treatment response (e.g., [48–50]). For example, by using the method of DeRubeis and colleagues, it was investigated in an effectiveness study among BPD patients which of two different treatments (DBT and General Psychiatric Management; GPM) would have been the optimal treatment option for a particular patient in terms of long term outcome [45]. The authors found that BPD patients with childhood emotional abuse, social adjustment problems, and dependent personality traits were more likely to benefit from DBT compared to GPM, whereas GPM excelled for patients with more severe problems related to impulsivity. The authors also provided an estimate of the advantage that might be gained if patients had been allocated to the optimal treatment option. The average difference in outcomes between the predicted optimal treatment and non-optimal treatment for all patients was small-to-medium (*d* = 0.36), while the advantage for patients with a relatively stronger prediction increased to a medium-to-large effect (*d* = 0.61). This suggests that treatment allocation based on a treatment selection procedure may substantially improve outcomes for BPD patients.

### Mechanisms of change

Another principal way to improve treatment response is to capitalize on mechanisms underlying change in treatments [27, 45, 51, 52]. Studying mechanisms of change helps to identify core ingredients of interventions and points the way to enhancing crucial elements, while discarding redundant elements. Presumably, this would maximize (cost-)effectiveness and efficiency as well. Since the 1950s, research on change processes has increased exponentially [53]. However, the majority of the trials on BPD have focused on outcomes, and only a few addressed *how* treatments exerted a positive effect on patient outcomes [54, 55]. Rudge et al. [56] reviewed studies on mechanisms of change in DBT. They concluded that there is empirical support for behavioral control, emotion regulation, and skills use as mechanisms underlying change in DBT. Recently, Yakın et al. [57] examined schema modes as mechanisms of change in ST for cluster C, histrionic, paranoid, and narcissistic personality disorders. They found that a strengthening of a functional schema mode (i.e., healthy adult mode) and weakening of four maladaptive schema modes (i.e., vulnerable child mode, impulsive child mode, avoidant protector mode, and self-aggrandizer mode) predicted improvements in PD symptomatology. However, changes in these schema modes, except for self-aggrandizer mode, also predicted improvements in outcome in treatment-as-usual and clarification-oriented psychotherapy, suggesting that modifying the strength of schema modes might reflect common mechanisms of change. The question of specificity of mechanisms of change is interesting, especially since both DBT and ST have their roots in cognitive behavior therapy and show similarity in certain treatment parameters, but differ substantially in techniques, explanatory model, and terminology [58]. Clarifying the treatment-specific and non-specific mechanisms of change may be key to furthering the effectiveness of both DBT and ST, and potentially also for psychotherapy in general.

### Current study

BPD-tailored treatments, like DBT and ST, are considered treatments of choice for BPD [25]. However, knowledge on the comparative (cost-)effectiveness of DBT and ST is lacking, as is knowledge on mechanisms of change and patient characteristics that predict (differential) treatment response. We will therefore perform a multicenter randomized clinical trial (RCT) comparing DBT and ST for BPD patients to elucidate the question “Which treatment – DBT or ST – works the best for which BPD patient, and why?”. The main aim of the BOOTS (Borderline Optimal Treatment Selection) study is to improve treatment response of DBT and ST for BPD patients by optimizing treatment selection through the identification of a prediction model based on patient characteristics that predict (differential) treatment response. By doing so, this study is a first step into the development of a treatment selection procedure for BPD patients. Moreover, the results of this study can serve as a starting point for future studies with the ultimate goal of implementing a treatment selection procedure that can be used in clinical practice to guide BPD patients and clinicians in selecting the optimal treatment. In addition, we aim to elucidate the mechanisms by which DBT and ST lead to change, thus pursuing the other main avenue towards improving BPD treatments.

This study has four primary objectives. The first objective of this study is to develop a treatment selection model based on a combination of patient characteristics that predict (differential) treatment response across DBT and ST. Candidate predictors of (differential) treatment response have been selected based on the literature, suggestions of a patient representative of the Borderline Foundation of the Netherlands, and clinicians’ appraisals of BPD patient characteristics that predict (differential) treatment response across DBT and ST. Semi-structured interviews were conducted among 18 expert clinicians to identify patient characteristics they deemed predictive of (differential) treatment response. The extensive investment in the identification of pertinent predictors is a lesson learned from Meehl [34], who noted that actuarial methods will not outperform clinical judgment when the actuarial method is based on inadequate knowledge of relevant variables. According to Westen and Weinberger [59], clinical expertise can serve the important function of identifying relevant variables for use in research. In addition, the majority of studies examining predictors of treatment response are based on randomized controlled trials with a primary focus on treatment effectiveness [60], which could result in the preclusion of potentially relevant predictors due to the lack of instruments assessing these constructs [39, 61]. Moreover, findings in the literature may be affected by publication bias, since statistically significant predictors of treatment response are more likely to be published [46]. Therefore, candidate predictors of (differential) treatment response are not only based on the literature, but also on clinical expertise and experience-based knowledge. We hypothesize that a combination of multiple patient characteristics will predict and moderate treatment effectiveness of DBT and ST. Hypotheses on the effects of single patient characteristics will not be formulated as research among BPD patients often failed to find consistent prognostic factors, while research on prescriptive factors or a combination between factors is scarce. In addition, there was in general a lack of consensus between the 18 expert clinicians on patient characteristics predicting (differential) treatment response across DBT and ST.

Second, we aim to elucidate how DBT and ST exert their effect by gaining a better understanding of the mechanisms of change of DBT and ST. A first step towards more insight into mechanisms of change is the identification of mediators. Mediators are easily confused with mechanisms of change, despite important differences [62]. A mediator is an intervening variable (partly) accounting for the statistical relationship between the intervention and outcome, and might serve as a statistical proxy for a mechanism of change [63]. In this study, we will examine potential BPD-treatment-specific, BPD-treatment-generic, and non-specific mediators. Based on empirical research and the presumed mechanisms of change (e.g., [55–57]), we hypothesize that change in skills use and emotion regulation are the mechanisms underlying change in DBT, and that change in schema modes and beliefs are the mechanisms of change in ST (i.e., BPD-treatment-specific mechanisms of change). In addition, a therapeutic environment characterized by genuineness of the therapists and group members, safety, and equality is considered to be especially important for BPD treatment [64–67] and is, therefore, assumed to be a BPD-treatment-generic mechanism of change. Finally, attachment and therapeutic alliance are the presumed non-specific mechanisms of change [68, 69].

Third, the comparative effectiveness of DBT and ST will be examined. Accumulating evidence suggests that symptoms and psychosocial functioning are only loosely associated [70, 71]. Patients with BPD are characterized by significant impairments in vocational functioning, relationships, and leisure [72]. In addition, social adjustment of BPD patients is considerably lower than social adjustment seen in other mental disorders, such as major depressive disorder and bipolar I disorder [73]. Moreover, although several studies found that even as psychopathology after treatment of BPD decreased, impairments in quality of life and functioning often (partly) persist [74, 75]. A more comprehensive view of recovery is therefore needed. This notion is underscored by qualitative research that has shown that patients define recovery by personal well-being, social inclusion, and satisfaction with life [76, 77]. Therefore, the current trial will track outcomes in multiple domains including symptoms, functioning, and well-being.

Finally, the cost-effectiveness of DBT and ST will be compared. Individual ST seems a cost-effective treatment [78, 79]. However, although group ST combined with individual ST is widely used in clinical practice, the cost-effectiveness of this combined program is yet unknown. An international RCT evaluating the (cost-)effectiveness of group ST for BPD is currently in progress [80]. More economic evaluations of DBT are available and support the cost-effectiveness of DBT. However, the studies vary highly in their design and the number of trials is still somewhat limited [26, 81, 82]. Therefore, an economic evaluation will be performed and a societal perspective will be applied, including indirect and direct healthcare costs.

In addition to these primary objectives, several secondary investigations will be performed, including (but not limited to): 1) the heterogeneity of BPD, 2) substance use (disorders) among patients with BPD, 3) perspectives of patients and therapists in key areas, including predictors, mechanisms of change, the treatments, and the implementation of the results in clinical practice, and 4) psychometric evaluations of several Dutch questionnaires (e.g., Dialectical Behavior Therapy-Ways of Coping Checklist, Ultrashort BPD Checklist).

## Methods/design

### Design

**Fig. 1:**
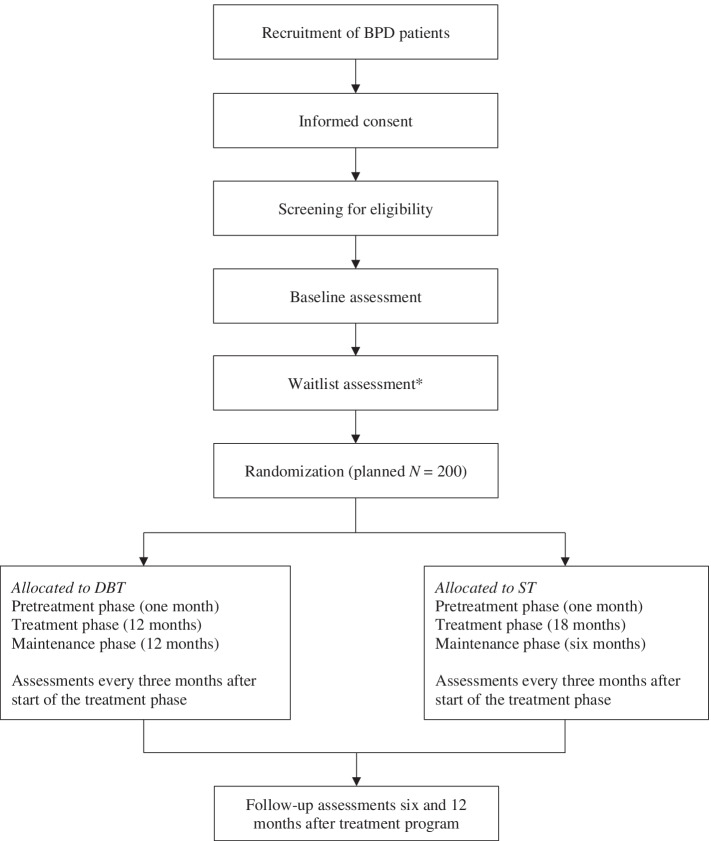
Flow chart of the study design. DBT = Dialectical Behavior Therapy; ST = Schema Therapy. *An extra assessment after wait is included for patients with a waitlist period of more than three months after the baseline assessment

The study is a multicenter RCT with two active conditions (DBT or ST). The study is set at various Dutch mental healthcare centers accessible through the public health system, including Antes (Rotterdam), GGZ inGeest (Amsterdam), GGZ NHN (Heerhugowaard), GGZ Rivierduinen (Leiden), NPI (Amsterdam), Pro Persona (Ede and Tiel), PsyQ (Rotterdam-Kralingen), and PsyQ/i-psy (Amsterdam). For an overview of the study design, including the enrollment, randomization, interventions, and assessments, see Fig. 1.

The Medical Ethics Committee of the Academic Medical Center (MEC-AMC) Amsterdam approved the study protocol (registration number NL66731.018.18). The study is registered at the Netherlands Trial Register, part of the Dutch Cochrane Center (registration number NL7699), and complies with the World Health Organization Trial Registration Data Set. Modifications to the protocol require a formal amendment to the protocol which will be examined by the MEC-AMC. The trial adheres to the SPIRIT methodology and guidelines [83], see Additional file 1.

### Patients

Patients are eligible if they 1) are between 18 and 65 years old, 2) have a primary diagnosis of BPD (diagnosed with the Structural Clinical Interview for DSM-5 Personality Disorders; SCID-5-PD), 3) have a BPD severity score > 20 on the Borderline Personality Disorder Severity Index, version 5 (BPDSI-5), 4) have an adequate proficiency in the Dutch language, and 5) are motivated to participate in (group) treatment for a maximum of 25 months and are willing and able to complete the assessments over a period of three years. Patients will be excluded if they 1) fulfill the criteria of a psychotic disorder in the past year (diagnosed with the Structural Clinical Interview for DSM-5 Syndrome Disorders; SCID-5-S), 2) have current substance dependence needing clinical detoxification, 3) have been diagnosed with a bipolar I disorder with at least one manic episode in the past year, 4) have been diagnosed with antisocial personality disorder (diagnosed with the SCID-5-PD), in combination with a history of physical violence against multiple individuals in the past two years, 5) have an IQ below 80, 6) have a travel time to the mental healthcare center longer than 45 min (except when the patient lives in the same city), 7) have no fixed address, and 8) have received ST or DBT in the past year.

### Sample size

We aim to include 200 participants. Each center intends to recruit at least 18 patients. For the power analysis, we adopted the minimal statistically detectable effect approach [84]. A sample size of 200 will be sufficient to have 80% power to detect moderators of treatment effects that have an effect size of Cohen’s *f* of .20 (small to medium effect size), based on a two-tailed significance level of *p* < .05. In addition, the study has 80% power to detect medium effect-sized (i.e., Cohen’s *f* = .25) moderators of treatment effects, based on a two-tailed significance level of *p* < .01.

Regarding the effectiveness study, with a sample size of *N* = 200 the study is powered at 82% to detect a group difference with a medium effect size of Cohen’s *d* = .50 at a two-tailed significance level of *p* < .05 and assuming a model with center as random effect and an intraclass correlation value of 0.05 corresponding to the center by treatment interaction [85, 86].

Finally, a sample size of *N* = 200 will be sufficient to have 98% power to detect a medium effect size of the mediation effect (*rr* = .09; [87–89]), assuming path *a* (relation between the predictor and mediator) and path *b* (relation between the mediator and outcome measure) both have a medium effect size (*r* = .30), and based on a simplified trivariate mediation model [90].

### Recruitment

Patients are recruited in the respective participating mental healthcare centers. Patients diagnosed with BPD or for whom this is deemed likely are invited to participate in the screening process. After reading and hearing information about the study and signing an informed consent (see Additional file 2, Appendix A), patients will start with the screening process. Not only new referrals can be included, but also patients who are already receiving treatment for mental disorders (except patients receiving ST or DBT).

### Randomization

A central independent research assistant randomizes the patients per center after a final check of the inclusion and exclusion criteria, and after all baseline measures have been completed. Generally, patients will be randomized using computerized covariate adaptive randomization [91–93], taking into account gender and severity of BPD (BPDSI-5 score ≤ 24; BPDSI-5 score > 24). By using this method, the imbalance of baseline characteristics between the treatments will be minimized. Patients are allocated to the treatment group that results in the least imbalance between the treatments with an allocation probability of 0.8 to preserve unpredictability [94]. Groups in both treatments are semi-open which implies that new patients can enter the group if treatment slots are available. Therefore, treatment capacity will be taken into account by using unequal ratios if needed (e.g., 2:1 or 1:3).

In exceptional cases, an alternative randomization method will be used if one or more treatment slots are available in only one condition and there is no available treatment slot in the other condition. To prevent long waiting times for treatment and empty places in the groups, the available treatment slot(s) in one condition will be randomized over 2*k patients whereby *k* stands for the number of available treatment slots, and randomization is done in the subsample of *k* patients that wait the longest. Randomization over 2*k patients guarantees unpredictable outcomes. For example, if one treatment slot is available in DBT and there is no available treatment slot in ST at that moment, nor within the foreseeable future, the available treatment slot in DBT will be randomized over two patients waiting for treatment. Sensitivity analyses will be performed by excluding patients that have been randomized using the alternative randomization method.

### Procedure and assessments

Patients with BPD or suspected of BPD are invited to the screening process by the research assistant or intake staff member. After providing written informed consent, patients are assessed for eligibility to participate in the study based on the inclusion and exclusion criteria. First, to assess DSM-5 syndrome disorders, the SCID-5-S is administered. The SCID-5-PD will also be administered in case the SCID-5-PD is not part of the standard intake procedure of the mental healthcare center. Second, the BPDSI-5 and a screening interview to assess the motivation and availability of the patient are conducted. A simple “yes” answer to the questions posed by the interviewer (e.g., “Are you motivated and available for treatment, including individual and group sessions?”) is not sufficient. Patients need to elaborate on their answers and follow-up questions are asked if needed. Patients who are eligible for participation will be invited for the baseline assessment, including interviews and computer-based self-report questionnaires, and intake staff members will fill out a questionnaire (i.e., intake questionnaire; see the Measures section) about these patients. After completing the baseline assessment, patients will be randomized as soon as treatment slots become available. Patients will be informed that they have been allocated to one of the treatment conditions, but the name of the treatment will not be communicated to the patient until the first treatment session. If patients cannot be randomized within several months after completing the baseline assessment because of unavailability of treatment slots, the BPDSI-5 will be re-assessed after three months and the BPDSI-5 and cost interview will be re-assessed after six months.

After the treatment phase has started, patients are reassessed every six months during the two years of treatment. These assessments are a combination of interviews and computer-based self-report questionnaires. In addition, a selection of measures are also assessed every three months, by computer-based self-report questionnaires. After end of the treatment, two follow-up assessments (six and 12 months after end of the treatment) will be administered. An overview of the measures is presented in Table 1. Candidate predictors of (differential) treatment response that are assessed only once at baseline are not included in Table 1. These measures can be found in the Measures section.

**Table 1:**
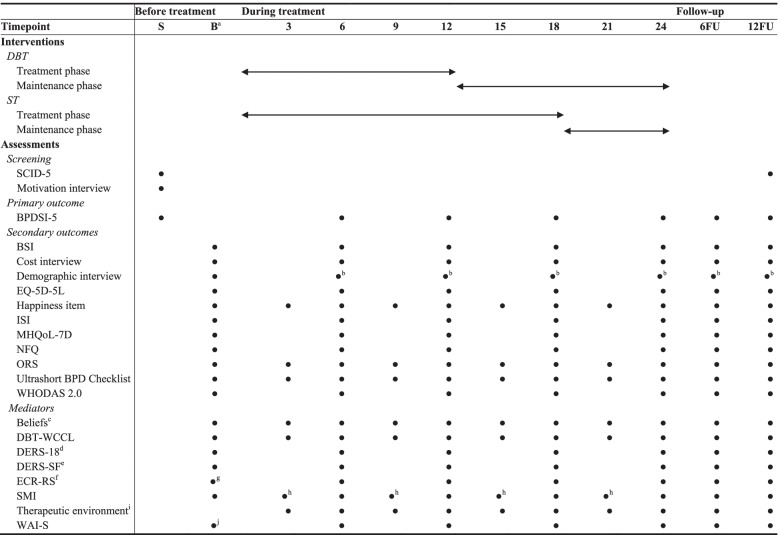
Overview of instruments

*S* Screening, *B* Baseline assessment; 3/6/9/12/15/18/21/24 = 3/6/9/12/15/18/21/24 months after start of the treatment phase, *6 FU* Follow-up at 6 months after end of treatment, *12 FU* Follow-up at 12 months after end of treatment, *BPDSI-5* Borderline Personality Severity Index, fifth edition, *BPD* Borderline personality disorder, *BSI* Brief Symptom Inventory, *DBT-WCCL* Dialectical Behavior Therapy-Ways of Coping Checklist, *DERS-18* Difficulties in Emotion Regulation Scale 18, *DERS-SF* Difficulties in Emotion Regulation Scale Short Form, *ECR-RS* Experiences in Close Relationships-Relationship Structures questionnaire, *EQ-5D-5L* 5-level EuroQol 5D version, *ISI* Insomnia Severity Index, *MHQoL-7D* Mental Health Quality of Life seven-dimensional Questionnaire, *NFQ* Nightmare Frequency Questionnaire, *ORS* Outcome Rating Scale, *SCID-5* Structured Clinical Interview for DSM-5, *SMI* Schema Mode Inventory, *WAI-S* Working Alliance Inventory-Short, *WHODAS 2.0* World Health Organization Disability Assessment Schedule 2.0

^a^The potential predictors of (differential) treatment response that are assessed only once at baseline are not included in this table

^b^A shortened version

^c^Including three to five idiosyncratic dysfunctional beliefs and one functional belief

^d^DERS-18 subscale ‘Awareness’

^e^Excluding the subscale ‘Awareness’

^f^Three versions of the ECR-RS will be assessed, measuring general attachment style and attachment styles with respect to two targets (i.e., most important therapist and group members)

^g^Two versions, measuring attachment styles with respect to two targets (i.e., most important therapist and group members), are assessed after the third group session

^h^SMI subscales Vulnerable Child, Angry Child, Impulsive Child, Detached Protector, Punitive Parent, and Healthy Adult

^i^Key characteristics of a promoting therapeutic environment (i.e., genuineness, safety, and equality) are assessed by 13 items formulated by ST experts

^j^Assessed after the third group session

All assessments are performed by trained local research assistants blind to the patients’ treatment condition, with exception of the SCID-5 interviews, demographic interview, and cost interview. The SCID-5 interviews can be administered by trained research assistants as well as trained intake staff members, both blind for condition. The demographic interview and cost interview contain questions on healthcare utilization and are therefore performed by non-blinded local research assistants. Due to the nature of the interventions, blinding of therapists and patients is not possible. All interviews, except for the SCID interviews, are audio-recorded. Participants receive financial compensation for their involvement in the study. Patients who discontinue their treatment or deviate from the treatment protocol will be encouraged to continue the assessments.

### Treatments

#### Format

For patients of both DBT and ST, treatment has a maximum duration of 25 months and starts with a pretreatment phase of approximately four weeks consisting of several (ST: ± three; DBT: ± five) individual sessions in which patients are prepared for the group sessions and become accustomed to their therapists and the treatment model. After the pretreatment phase, patients receive a combined program of individual sessions and group sessions (i.e., treatment phase). Group sessions of both treatments are offered in a semi-open format. If treatment slots are available, new patients can enter the ST group every 10 weeks and for DBT groups at the start of a mindfulness skills module. In DBT, the treatment phase has a maximum duration of 12 months and consists of weekly group sessions (i.e., skills training groups; 150 min), weekly individual psychotherapy sessions (50 min), and between-session consultation. The between-session consultation, often called telephone consultation although all kinds of technology can be used [95], is offered to the patient within limitations set by the individual therapist, varying between access to between-session support within working hours to 24/7 access to between-session support, which is officially the standard in DBT. In ST, the treatment phase has a maximum duration of 18 months consisting of weekly group (90 min) and individual (45 min) psychotherapy sessions for a period of 12 months, continued by weekly group psychotherapy sessions and biweekly individual psychotherapy sessions for a period of six months. Following the treatment phase, patients continue their treatment with a maintenance phase. The maintenance phase of DBT is a recently developed blended aftercare program with a maximum duration of 12 months. The blended aftercare program was developed based on results of previous studies (e.g., [31, 96]) and recommendations by several authors (e.g., [96–98]) to extend the duration of DBT to sustain or even enhance treatment effects. The DBT aftercare program consists of monthly individual psychotherapy sessions, three-monthly group sessions, and an eHealth intervention in which patients have online access to DBT handouts and worksheets [99]. The maintenance phase of ST consists of biweekly individual psychotherapy sessions for a period of three months, continued by three months of one individual session each month. Disregarding the time spent on telephone consultation, homework assignments, and eHealth, and based on 48 working weeks a year, patients will receive about 167 h of treatment if they follow the treatment protocol. Patients who have completed treatment successfully before they reach the maximum number of treatment sessions are allowed to complete treatment earlier, although the assessments will be conducted at the originally planned assessment points. Early termination of treatment requires substantial improvements in the primary and secondary outcomes and is decided in joint decision by the patient and therapist. The treatments are covered by the public health insurance. See Table 2 for an overview of the treatment formats.

**Table 2:** Treatment formats

ST	Duration	DBT	Duration
Pretreatment phase	4 weeks	Pretreatment phase	4 weeks
Individual sessions		Individual sessions	
Treatment phase	18 months	Treatment phase	12 months
Weekly individual sessions	12 months	Weekly individual sessions	
Weekly group sessions		Weekly skills training groups	
Biweekly individual sessions	6 months	Telephone consultation	
Weekly group sessions			
Maintenance phase	6 months	Maintenance phase	12 months
Biweekly individual sessions	3 months	eHealth intervention	
Monthly individual sessions	3 months	Monthly individual sessions	
		Three-monthly group sessions	

Note. Early termination of treatment during the treatment phase or maintenance phase is permitted in case of successful recovery

#### Schema therapy (ST)

ST, developed by Jeffrey Young [22, 100], is based on an integrative cognitive model, combining cognitive behavior therapy and experiential techniques with insights from developmental theories, including attachment theory, and psychodynamic concepts [23]. Central concepts are early maladaptive schemas and schema modes. Early maladaptive schemas can be defined as broad, pervasive patterns of thoughts, emotions, memories, and cognitions regarding oneself and relationships with others, developed during childhood [22]. ST assumes that the frustration of core needs and early traumatic experiences lead to the development of early maladaptive schemas. A schema mode refers to an activated set of schemas and the associated coping response (i.e., overcompensation, avoidance, and surrender), and describes the momentary emotional, cognitive, and behavioral state of the patient. The following schema modes are characteristic of BPD [101]: 1) vulnerable child mode, associated with a fear of abandonment and strong emotions, such as loneliness, sadness, and helplessness, 2) angry and impulsive child mode, characterized by anger, frustration, hostility, and impulsivity, 3) punitive parent mode, representing the internalized voice of very punitive and critical attachment figures and associated with self-criticism, self-hatred, guilt, and self-denial, 4) detached protector mode, characterized by attempts to cut off the self from needs and feelings, resulting in symptoms of detachment, substance misuse, social withdrawal, and self-harm, and 5) healthy modes, reflecting in functional thoughts, cognitions, and behavior (i.e., healthy adult mode) and the feeling that core needs are been fulfilled (i.e., happy child mode). The first four modes are maladaptive schema modes and central to BPD. The last two modes are functional and often only weakly present at the beginning of the treatment [102]. Idiosyncratic schema mode models usually cover additional modes, depending on the specific problems and comorbidity of the patient.

ST aims to enable patients to fulfill their needs, reduce maladaptive schema modes, and strengthen adaptive schema modes. In this study, ST is offered in a combined group-individual format developed by Farrell and Shaw [103]. The group acts as an analogue of a family with the other patients as “siblings” and the two therapists as “parents” [103]. The group may speed up and amplify the effect of treatment by offering corrective emotional experiences, peer support, opportunities for in vivo practice, and a sense of understanding [104]. The individual ST follows the protocol as described by Arntz and Van Genderen [105].

#### Dialectical behavior therapy (DBT)

DBT is a comprehensive cognitive behaviorally based treatment for BPD, integrating strategies from cognitive and behavioral treatments, Zen-based acceptance strategies, and dialectical strategies [19, 106]. Linehan [19, 20] proposed a skills deficit model in which emotion regulation is central. More specifically, the model holds that the problematic behaviors associated with BPD (e.g., suicide attempts, self-injury, substance use) are in fact best understood as dysfunctional attempts to regulate emotions. Emotion dysregulation results from the complex transaction between dispositional emotional vulnerabilities and an adverse invalidating environment. Therefore, the treatment involves balancing problem solving strategies with loads of validation. DBT aims to help patients develop new skills, enhance motivation, ensure generalization of skills use, and change their environment if needed. In addition, DBT aims to enhance therapists’ motivation to deliver effective treatment [20].

DBT involves skills training groups, individual therapy, between-session consultation, and therapist consultation team meetings. DBT skills training groups teach patients behavioral skills in four different, yet inter-related, areas: mindfulness, interpersonal effectiveness, emotion regulation, and distress tolerance / radical acceptance. Individual therapy focuses on motivational issues and the acquisition and use of skills in daily life. A predetermined ordering of treatment targets is used in individual sessions and part of different stages of the treatment. Stage 1 focuses on stabilizing the patient and behavior control. Targets in this stage of the treatment include: life-threatening behavior, therapy-interfering behavior, quality-of-life-interfering behavior, and behavior skills. Stage 2 focuses on reducing posttraumatic stress and requires exposure to trauma-related cues [19]. Finally, Stages 3 and 4 target self-respect and the sense of incompleteness. However, due to time constraints, some patients might not enter all stages and most studies have focused on Stage 1 DBT [107]. Individual therapists provide between-session (telephone) consultation if needed. According to the guidelines of DBT, access to between-session consultation outside of office hours, preferably by the individual therapist, is part of DBT [19]. In this trial, between-session consultation by the individual therapist will be within limitations set by the therapist, which can vary between support provided within working hours to 24/7 access to telephone consultation. As access to between-session (telephone) consultation will vary between centers and individual therapists, the effect of therapist’s availability for between-session support will be examined. Finally, DBT therapists meet weekly in a DBT consultation team to motivate and support each other.

#### Therapists, training, and supervision

The therapists in this study will be licensed psychologists, psychotherapists, psychiatrists, or psychiatric nurses. Individual and group schema therapists must have completed a basic training in individual ST. Group schema therapists must have also completed a four-day training in the group schema therapy model of Farrell and Shaw [103]. All schema therapists receive a one-day training in experiential techniques by a certified ST trainer. DBT therapists are required to complete a three-day training in DBT and at least one member of the DBT team must have completed the 10-day intensive DBT training. In addition, DBT therapists receive a two-day kick-off training by certified DBT trainers to expand their knowledge of DBT. Moreover, DBT-therapists were given the opportunity to participate in a one-day training in imaginal exposure. According to Linehan [19], reducing behaviors and stress response patterns related to traumatic life events is a primary DBT target. Reducing posttraumatic stress is mostly part of Stage 2 of DBT and involves exposure to trauma-associated cues [19, 108]. However, some of the DBT therapists expressed concerns about their ability to apply the principles and procedures of exposure to treat traumatic memories in BPD patients. Therefore, the opportunity to participate in a one-day exposure training was offered to the therapists.

Before the start of the study, schema therapists should have received at least 10 individual supervision sessions by a licensed supervisor. There is no requirement for the minimum number of DBT supervision sessions. During the study, therapists receive supervision over a period of two years by certified supervisors. ST supervision is provided through teleconferencing biweekly in the beginning, then (two-)monthly after six to 12 months, depending on the experience of the therapists. DBT therapists receive supervision at location every three months. Moreover, there will be weekly DBT team meetings (i.e., DBT consultation team meetings) and biweekly ST team meetings. All individual ST sessions will be audiotaped, while individual DBT sessions and ST and DBT group sessions will be videotaped. These recordings are used for supervision and treatment adherence ratings. Treatment adherence, a component of treatment integrity (i.e., the extent to which a treatment is implemented as intended; [109]), refers to the extent to which the therapist utilizes prescribed techniques and procedures and avoids the use of proscribed techniques and procedures [110]. Adherence will be assessed in a random selection of session recordings by trained raters (master psychology students) blind for condition. Master psychology students will be trained by ST and DBT experts by using session recordings not used in the final adherence rating to practice with the instruments. Individual ST sessions will be rated on an adapted version of the Therapy Adherence and Competence scale for ST for BPD [111] and group ST sessions will be rated on the Group Schema Therapy Rating Scale – Revised [112]. Individual DBT sessions will be rated on the Dutch translation of the observer-rated version of the DBT Adherence Checklist for Individual Therapy [113]. An observer-rated instrument will be developed to assess the skills training groups.

#### Other treatment

During the treatment, patients are not allowed to engage in any other form of psychological treatment. However, in case of acute crisis, the crisis procedures of the treatments will be followed (e.g., telephone consultation by the therapist, contact a crisis line, visit the emergency room, hospitalization, individual crisis management sessions). Any additional treatment will be recorded and included in the analyses. Patients will only be withdrawn from the study at their request.

### Coronavirus disease (COVID-19) pandemic

This study is conducted during the COVID-19 pandemic. The pandemic is expected to have adverse effects on patients with mental health disorders [114]. In addition, in case face-to-face treatment is restricted in mental healthcare centers because of government and healthcare center policy, the treatment will be delivered via videoconferencing. Consequently, differences between patients will arise regarding the amount of treatment sessions delivered during the pandemic and/or via videoconferencing. We will control for a potential influence of the COVID-19 pandemic by, for example, adding dynamic regression parameters that include the impact of time in treatment during the pandemic. The definition of the indicator variable indicating the COVID-19 pandemic will be decided before start of the data-analyses (e.g., dummy variable indicating pandemic/no pandemic or continuous variable indicating the amount of time in treatment during the pandemic), given the unpredictability of the current situation. Moreover, exploratory analyses may be conducted to investigate the potential influence of the deviating treatment format (i.e., online vs. face-to-face individual sessions and/or group sessions) on the treatment effectiveness.

In addition, the assessments will be conducted via videoconferencing or phone, and the computer-based questionnaires will be completed by participants at home, if face-to-face assessments are not allowed. Before receiving the treatment and/or assessments via videoconferencing, patients will sign an additional informed consent form (see Additional file 2, Appendix B).

### Data management, storage, monitoring, and dissemination

Data is collected with a unique identifier for each patient (i.e., pseudonym) using the online survey software program Qualtrics [115] and the web tool Lotus, which has been especially developed for longitudinal research by the University of Amsterdam. The list of pseudonyms and personal information of patients within a particular mental healthcare center is securely stored at the center and only accessible for the research assistant and coordinator of this center. A different set of pseudonyms is used for data collected by clinicians (i.e., intake questionnaire and recordings). The list with the combination of both pseudonyms of patients is only accessible for the research assistant and coordinator of the center and the authorized researchers. The data is stored on a secure storage server of the University of Amsterdam, accessible only to authorized researchers.

All (serious) adverse events reported by the patient or observed by clinicians or researchers will be recorded. There is no data monitoring committee and the study will not be audited. The results of the study will be disseminated in scientific journals and presentations at (inter)national scientific conferences.

### Measures

The instruments include screening measures, measures to assess potential predictors and mediators of treatment response, and outcome measures. The instruments that were not available in Dutch were translated (i.e., Brief Experiential Avoidance Questionnaire, Dialectical Behavior Therapy-Ways of Coping Checklist, Gordon Test of Visual Imagery Control, Positive Mental Health scale, and social problems) by bi-lingual experts. The translations were checked for consistency with the original version. Items, questionnaires, and interviews that have been developed or modified by the authors are available upon request by the first author.

#### Screening

##### Mental disorders

The SCID-5 is a semi-structured interview used to diagnose DSM-5 disorders. Personality disorders are assessed with the SCID-5-PD [116] and syndrome disorders are assessed using the SCID-5-S [117], which is an extended version of the SCID-5 Clinician Version (SCID-5-CV; [118]). Additional file 3 offers an overview of all syndrome disorders that are assessed by the SCID-5-S. Based on a first psychometric evaluation in a psychiatric patient sample, Somma et al. [119] found an adequate interrater reliability of the SCID-5-PD. In addition, the SCID-5-CV has demonstrated good psychometric properties [120–122].

Before administering the SCID-5-S and/or SCID-5-PD, self-report screening questionnaires (SCID-5-SPQ; [123], and SCID-5-SV; [124]) may be administered. In accordance with the instructions for administering the SCID, disorders and criteria of disorders not affirmed by the screening questionnaires and not considered as false negatives by the clinician will be assumed to be absent. The SCID-5 will be assessed during the screening phase and 12 months after end of the treatment.

##### Motivation and availability

A 13-item semi-structured motivation interview is used to assess several exclusion criteria (e.g., no fixed address, have received ST or DBT in the past year) and patient’s motivation and availability.

#### Predictors

As mentioned, candidate predictor variables of (differential) treatment response have been selected using a multi-method approach (i.e., literature, suggestions of a patient representative of the Borderline Foundation of the Netherlands, and semi-structured interviews with 18 expert clinicians). Additional file 4, Table 1 offers an overview of the predictors that have emerged during the semi-structured interviews with clinicians. Additional file 4, Table 2 offers an overview of the predictors based on the literature and suggestions of a patient representative. The candidate predictors of (differential) treatment response are assessed at baseline. Only the measures that are not part of the screening, mediator or outcome measures will be briefly described in this paragraph.

##### Autistic traits

Autistic traits are assessed by the abbreviated version of the Autism Spectrum Quotient, the AQ-10 [125]. The AQ-10 consists of 10 items rated on a four point Likert scale. The AQ-10 has demonstrated acceptable psychometric properties in an adult general population sample [126].

##### Commitment

Patient commitment to treatment is measured with a selection of items of the subscale Motivation to Engage of the Treatment Motivation Scales for forensic outpatient treatment (TMS-F; [127]). The four items can be rated on a seven point Likert scale.

##### Experiential avoidance

The Brief Experiential Avoidance Questionnaire (BEAQ; [128]) is a 15-item scale assessing experiential avoidance across six domains (i.e., behavioral avoidance, distress aversion, suppression, procrastination, repression/denial, and distress endurance). The items can be rated on a six point Likert scale. The BEAQ has shown good psychometric properties among psychiatric outpatients [128].

##### Frustration intolerance

Frustration intolerance is assessed by the Frustration Tolerance subscale of the Severity Indices of Personality Problems (SIPP-118; [129]). This subscale consists of eight 4-point Likert scale items measuring the capacity to cope with setbacks and disappointments. In previous research among Dutch patients with a personality disorder, the subscale demonstrated moderate to good reliability [129].

##### Insight

A modified version of the Self-Reflection and Insight Scale (SRIS; [130, 131]) is used to assess self-reflection and insight. The SRIS contains 20 five point Likert scale items. The SRIS has shown good reliability and validity in student samples [130, 132].

##### Internal locus of control

Internal locus of control, defined as the extent to which a person experiences an outcome as the result of their own behavior or personal characteristics rather than external circumstance, is assessed by the Locus of Control scale (IE; [133]). The IE contains 10 five point Likert scale items. Previous research has demonstrated adequate psychometric properties [133, 134].

##### Level of personality functioning

The Level of Personality Functioning Scale-Brief Form 2.0 (LPFS-BF 2.0; [135]) assesses impairment in personality functioning according to the DSM-5 alternative model for personality disorders. The LPFS-BF 2.0 contains 12 four point Likert scale items. Based on a first psychometric evaluation among Dutch patients referred to a specialized mental healthcare center for personality disorders, the LPFS-BF 2.0 has demonstrated satisfactory psychometric properties [135].

##### Mental imagery capacity

Mental imagery capacity is assessed with the 12-item Gordon Test of Visual Imagery Control (TVIC; [136]). The TVIC assesses the ability to visualize and manipulate a given scenario in response to a set of cues. Participants can response on a three point Likert scale. In addition to the 12 Likert scale items, we measure the time it takes the participant to visualize the scenarios. Finally, we have added two 100 mm visual analog scale (VAS) items measuring how well participants see the scenarios that were described and how difficult it was for the participant to visualize the different scenarios. The TVIC has demonstrated fair to satisfactory internal consistency and validity among community samples and undergraduates [137–140].

##### Mentalizing capacity

Mentalizing capacity is measured using an eight-item version of the Reflective Functioning Questionnaire (RFQ-8; [141]). The RFQ-8 comprises two dimensions: uncertainty about mental states, reflecting hypomentalizing, and certainty about mental states, indicating hypermentalizing. The RFQ-8 uses a seven point Likert scale. In previous research among BPD patients, the questionnaire has demonstrated satisfactory psychometric properties [141–143].

##### Perfectionism

The eight-item Frost Multidimensional Perfectionism Scale-Brief (F-MPS-Brief; [144]) assesses perfectionism across two dimensions (evaluative concerns and striving). Items are rated on a five point Likert scale. Psychometric properties of the F-MPS-Brief were found to be good in clinical and community samples [144].

##### Personality traits

Personality traits are measured, among others, with the Ten-Item Personality Inventory (TIPI; [145, 146]), which is a brief measure of the Big-Five personality dimensions. The 10 items can be rated on a seven point Likert scale. The TIPI has shown low to moderate internal consistency and adequate validity among students [145, 146].

##### Positive mental health

Positive mental health, often referred to as mental well-being, is assessed using the nine-item Positive Mental Health scale (PMH-scale; [147]). The items can be rated on a nine point Likert scale. Based on a previous study on the psychometric properties of the PMH-scale in student, patient and general samples, the PMH-scale was found to be a reliable and valid instrument [147].

##### Psychopathology and maladaptive personality traits

The Minnesota Multiphasic Personality Inventory-2 Restructured Form (MMPI-2-RF; [148]) measures a wide range of psychopathology symptoms, personality characteristics, and behavioral proclivities. The MMPI-2-RF consists of 338 true-false items aggregating onto 51 individual scales. The psychometric properties of the MMPI-2-RF varied from inadequate to good among normative, outpatient, and inpatients samples, as documented in detail in the Technical Manual [149].

##### Readiness to change

Readiness to change is assessed by two subscales (contemplation and action) of the 24-item version of the University of Rhode Island Change Assessment (URICA; [150–152]). Both subscales are measured by six 5-point Likert scale items and have demonstrated good reliability across a diversity of studies (e.g., [153–155]).

##### Rigidity

Rigidity is measured by the Rigidity subscale of the Computerized Adaptive Test of Personality Disorder-Static Form (CAT-PD-SF; [156]). The Rigidity subscale contains 10 five point Likert scale items reflecting an unwillingness to consider alternative perspectives and inflexibility in values and beliefs. The subscale has demonstrated good reliability among community adults with current or a history of mental health treatment [156].

##### Social problems

By using the social problems list, derived from the Improving Access to Psychological Therapies (IAPT) program [157], social problems (e.g., financial problems, housing problem, and unemployment) are assessed in direct discussion with the patient.

##### Social support

The Multidimensional Scale of Perceived Social Support (MSPSS; [158]) is assessed to investigate perceived support from three sources: significant others, family, and friends. The MSPSS contains 12 items which can be rated on a seven point Likert scale. Psychometric properties of the MSPSS are satisfactory among psychiatric outpatients and BPD patients [159, 160]. In addition to the MSPSS, the research assistant rates the patient’s social network taking into account the size of the network and potential pathogenic influences.

##### Stigma of immutability

BPD has been associated to stigma of immutability [161]. We have developed five 7-point Likert scale items assessing the extent to which participants believe that BPD is resistant to treatment.

##### Trauma

The Traumatic Experience Checklist (TEC; [162]) is used to assess traumatic experiences, including emotional abuse, emotional neglect, sexual abuse, sexual harassment, physical abuse, and threat to life/ bizarre punishment/ intense pain. The TEC includes 30 descriptions of various traumatic experiences. The TEC has demonstrated favorable psychometric properties in Dutch psychiatric patients [162].

##### Verbal intelligence

The Dutch version of the National Adult Reading Test (DART; [163]) is used as a proxy for verbal intelligence. The DART is a reading test including 50 irregularly spelled words. Based on previous research, the DART yields an adequate estimation of verbal intelligence and has shown adequate psychometric properties across a variety of populations [164].

##### Other patient characteristics, collected using a self-report questionnaire

In addition to the questionnaires, participants fill out several questions developed by the authors about the willingness and ability to engage in a therapeutic relationship, perceived suitability of DBT and ST (treatment preference), and the absence or presence of an attachment figure in the past.

##### Other patient characteristics, collected using a questionnaire filled out by clinicians (intake questionnaire)

Clinicians responsible for the intake assessment will fill out the nine-item intake questionnaire for each participant, including questions about the willingness and ability to engage in a therapeutic relationship, the willingness and ability to examine the link between childhood history and present problems, high vs. low level borderline personality organization [165], the request for help, the degree to which a syndrome disorder might interfere with treatment response, and perceived suitability of DBT and ST. These questions have been formulated by the authors.

#### Mediators

Both treatments include non-specific (attachment and therapeutic alliance), BPD-treatment-generic (therapeutic environment characterized by genuineness, safety, and equality), and BPD-treatment-specific (ST: beliefs and schema modes; DBT: emotion regulation and skills use) mechanisms of change. The proposed mediators are repeatedly measured: at baseline, except for measures requiring information about the therapy (i.e., therapeutic environment, therapeutic alliance, and attachment styles with respect to the most important therapist and group members), and every six months after start of the treatment phase. In addition, a selection of the proposed mediators (i.e., selection of schema modes, skills use, beliefs, and therapeutic environment) are also collected every three months after start of the treatment phase, during the first two years.

##### Attachment

The Experience in Close Relationships-Relationship Structures Questionnaire (ECR-RS; [166]) is a brief version of the Experience in Close Relationships-Revised (ECR-R; [167]). The ECR-RS measures attachment patterns in different relational domains, such as relationships with parents and friends. The ECR-RS can also be adapted to measure a person’s general attachment style. In this study, three versions of the ECR-RS are used, measuring general attachment style and attachment styles with respect to two targets (i.e., most important therapist and group members). The ECR-RS contains nine items, assessing two attachment dimensions: attachment-related anxiety and avoidance. The items can be rated on a seven point Likert scale. The ECR-RS has shown adequate psychometric properties in a large web-based sample (*N* > 21.000), comparable to the ECR-R [166]. As experience with the treatment is required in order to be able to complete the questions about the most important therapist and group members, these questions will be filled out three weeks after start of the treatment phase.

##### Beliefs

Idiosyncratic dysfunctional beliefs were elicited with a semi-structured interview at baseline. Three to five idiosyncratic dysfunctional beliefs related to the self (e.g., “I am worthless”), others (e.g., “People always reject me”), and emotions (e.g., “Expressing emotions is a sign of weakness”) are formulated. Participants rate the degree to which they believe in each statement on a 100 mm VAS at baseline and at every subsequent assessment. This procedure has been used in previous research (e.g., [168, 169]). The VAS has found to be useful for assessing variations in intensity of beliefs in patients with a personality disorder [169]. In addition to the idiosyncratic dysfunctional beliefs, participants rate the credibility of one functional belief (“I consider myself a good person”) on a 100 mm VAS.

##### Emotion regulation

Emotion regulation is assessed by the Difficulties in Emotion Regulation Scale Short Form (DERS-SF; [170]), a brief version of the widely used DERS [171]. The DERS-SF measures non-acceptance of emotional responses, difficulties engaging in goal-directed behavior, impulse control difficulties, limited access to emotion regulation strategies, lack of emotional clarity, and lack of emotional awareness. The awareness subscale is excluded based on recommendations of among others Hallion et al. [172] and Bardeen et al. [173]. Lack of emotional awareness is assessed by the Awareness subscale of the Difficulties in Emotion Regulation Scale 18 (DERS-18; [174]). The DERS-SF, without the awareness subscale, consists of 15 items. The Awareness subscale of the DERS-18 is measured by three items. All items can be rated on a five point Likert scale. Both questionnaires have demonstrated good psychometric properties among outpatients [172].

##### Schema mode ratings

The Schema Mode Inventory (SMI; [175]) measures the extent to which 16 different (dysfunctional as well as functional) schema modes are endorsed. The SMI consists of 143 items that are scored on a six point Likert scale. Previous research using a sample of non-patients and patients with a syndrome disorder and/or personality disorder has demonstrated acceptable psychometric properties [176]. The five maladaptive schema modes that are central to BPD (i.e., vulnerable child, angry child, impulsive child, detached protector, and punitive parent; [101]) and one functional schema mode (i.e., healthy adult) are assessed every three months during the first two years.

##### Skills use

The 59-item Dialectical Behavior Therapy-Ways of Coping Checklist (DBT-WCCL; [177]) is an adaptation of the Revised Ways of Coping Checklist (RWCCL; [178]). The DBT-WCCL measures DBT skills use and maladaptive coping skills use over the previous month. All items are assessed using a four point Likert scale. The DBT-WCCL has shown adequate to excellent reliability and validity among BPD patients [177].

##### Therapeutic alliance

The therapeutic alliance is measured with the Working Alliance Inventory-Short (WAI-S; [179, 180]). The WAI-S consists of three subscales (agreement on goals, agreement on tasks, and bond between patient and therapist), each consisting of four items which can be scored on a five point Likert scale. Observed psychometric properties of the WAI-S were satisfactory in a patient sample [179, 181]. Since experience with the treatment is required in order to be able to complete the WAI-S, the WAI-S will be filled out three weeks after start of the treatment phase.

##### Therapeutic environment

Key characteristics of a promoting therapeutic environment (i.e., genuineness, safety, and equality) are assessed by 13 items formulated by ST experts (A. Arntz and O. Brand-de Wilde) and rated on a 100 mm VAS. The items measure the extent to which the participant feels a) the individual therapist, group therapists, and group members are genuine with him/her; b) he or she can tell the individual therapist and group therapists everything; c) safe in the individual and group therapy; d) safe to show vulnerability and express negative feelings in the individual and group therapy; e) the individual and group therapists take personal responsibility for their mistakes; and f) the individual and group therapists see him/her as equal. Since experience with the treatment is required in order to be able to complete this questionnaire, this questionnaire will not be assessed at baseline.

#### Primary outcome

##### BPD severity

The primary outcome measure is the change in severity and frequency of the DSM-5 BPD manifestations between baseline until three-year follow-up, assessed with the total score of the Borderline Personality Disorder Severity Index version 5 (BPDSI-5; [182, 183]). The BPDSI-5 is a semi-structured interview consisting of 70 items rating the nine DSM-5 BPD criteria over the prior three months. All items are rated on a 11-point Likert scale (0 = never to 10 = daily), except for the subscale Identity Disturbance which is rated on a 5-point Likert Scale (0 = absent to 4 = dominant, clear, and well-defined) and multiplied by 2.5. The total score consists of the sum of the nine criteria scores and ranges from 0 to 90. The scores on the BPDSI-5 subscales provide information on the severity of each of the nine criteria. The BPDSI-5 is a modified version of the BPDSI-IV [182, 183] in which a few questions have been slightly reworded and exact frequency scores have been added in addition to the Likert scale. The BPDSI-IV has proven to be a reliable and valid measure among non-patients and (BPD) patients [182, 183]. Previous research has shown that a cut-off score of 15 differentiates between BPD patients and controls [183]. In addition, a score of 20 distinguishes BPD patients from non-BPD patients [183–185].

#### Secondary outcome measures

As accumulating evidence suggests that BPD severity and level of functioning are only loosely associated, attention will be paid to outcomes in different areas, including symptoms, functioning, and well-being. The outcome measures are administered at baseline and every six months after start of the treatment phase. In addition, patients’ ratings of experienced burden due to BPD manifestations and well-being are collected every three months after start of the treatment phase, during the first two years.

##### Costs

Costs, including healthcare costs, patient and family costs, and costs outside the healthcare sector, are measured using a retrospective cost interview especially designed for BPD patients [80]. Healthcare costs include visits to general practitioners, hospitals, crisis centers, psychologists and psychiatrists, use of medication, social work, paramedical care, and alternative treatments. Patient and family costs include informal care (i.e., care provided by the patient’s family, friends, or neighbors) and out of pocket costs (e.g., drugs, alcohol, excessive spending). Costs in other sectors include productivity losses from unpaid work (study and voluntary work) and paid work. Since it is difficult to distinguish between BPD-related costs and costs due to other psychological disorders [17], only a distinction will be made between costs due to psychological disorders and costs due to somatic diseases. The cost interview will be conducted by trained research assistants using a recall period of six months (baseline assessment), the number of weeks since randomization (assessment six months after start of the treatment phase), or the number of weeks since the previous assessment (assessments 12, 18, and 24 months after start of the treatment phase and both follow-up assessments).

Dutch guidelines [186, 187] will be used to determine total costs. Healthcare costs will be calculated by volumes of resource use multiplied by their corresponding unit costs, derived from Hakkaart-van Roijen et al. [186]. Prescribed medication costs will be determined based on national reference prices. Informal care costs will be computed by multiplying the number of hours the patient receives informal care by shadow prices [186]. Shadow prices will also be used to value lost productivity in study and voluntary work. Productivity losses from paid work will be valued according to the Human Capital Approach [188]. Out of pocket costs, such as alcohol and excessive spending, will be directly retrieved from the cost interview or, in case of over-the-counter medication, from the Dutch Pharmacotherapeutic Compass [186].

##### Demographics

General patient characteristics (e.g., age, ethnicity, marital status, educational level, employment status) will be collected using a semi-structured demographic interview. During this interview, additional patient characteristics such as treatment history, request for help, medication use, substance use, and duration of BPD manifestations will be recorded. For an overview of all characteristics, see Additional file 4.

##### Experienced burden due to BPD

Patient’s self-reported experienced burden of BPD manifestations are measured using the Ultrashort BPD Checklist, a shortened version of the validated BPD Checklist [189]. The Ultrashort BPD Checklist consists of nine to 11 5-point Likert scale items (the number of items will be based on the upcoming validation study), each related to a specific DSM-5 BPD criterion. Based on an initial psychometric evaluation, the Ultrashort BPD Checklist showed good to excellent psychometric properties in a sample with BPD and cluster C patients, patients with a syndrome disorder, and non-patients, similar to the BPD Checklist [189].

##### General psychopathological symptoms

The Brief Symptom Inventory (BSI; [190, 191]) is a self-report instrument measuring general psychiatric symptoms at the time of assessment. The BSI is a short version of the Symptom-Check-List (SCL-90-R) and contains 53 items assessing nine symptom dimensions: somatization, obsession-compulsion, interpersonal sensitivity, depression, anxiety, hostility, phobic anxiety, paranoid ideation, and psychoticism. All items are assessed using a five point Likert scale. Previous research in Dutch community and patient samples has demonstrated good reliability and validity [191, 192].

##### Global functioning and impairment

Global functioning and impairment is assessed by the 36-item World Health Organization Disability Assessment Schedule 2.0 (WHODAS 2.0) interview version [193]. The WHODAS 2.0 is a general measure to assess disability in six major life domains (cognition, mobility, self-care, getting along, life activities, and participation). For each item, participants have to report how much difficulty they experienced in the last 30 days. The six domain scores and overall functioning score have shown good psychometric properties in a general population sample as well as a patient sample [193].

##### Quality of life

Generic quality of life is assessed using the 5-level EuroQol 5D version (EQ-5D-5L; [194]). The questionnaire measures five health state dimensions (mobility, self-care, usual activities, pain/discomfort, and anxiety/depression). Each dimension is divided into five severity levels: no problem, slight problems, moderate problems, severe problems, and extreme problems. The profiles from the five health state dimensions are assigned a value based on the Dutch social tariffs to generate health utilities [195]. These utilities will be used to calculate Quality Adjusted Life Years (QALYs) by multiplying the change in utility values between assessments by the length of the period between assessments. In addition to the five health state dimensions, the EQ-5D-5L contains a VAS item which records the patient’s self-reported health status ranging from 0 (worst health you can imagine) to 100 (best health you can imagine). The EQ-5D-5L has shown to be a reliable and valid measure among different patient groups in different countries [196].

As a complement to the EQ-5D-5L, the Mental Health Quality of Life seven-dimensional Questionnaire (MHQoL-7D; [197]) will be administered. The MHQoL-7D is a recently developed instrument to assess quality of life specifically in people with mental health problems. The MHQoL-7D consists of seven quality of life domains (self-image, independence, mood, relationships, daily activities, physical health, and hope) and a VAS item which records the patient’s self-reported psychological well-being. A study into the psychometric properties of the MHQoL-7D is currently running. The MHQoL-7D will only be included in the analysis if it is demonstrated to be a psychometrically sound instrument and Dutch social tariffs are available.

##### Sleep

Insomnia complaints are assessed by the Insomnia Severity Index (ISI; [198]). The ISI contains seven items that are scored on a five point Likert scale. The ISI has shown to be a valid measure in community and insomnia patient samples [198], although the reliability was questionable in some studies (e.g., [199, 200]). In addition to insomnia, the number of nights with nightmares and the total number of nightmares in the week prior to the assessment are measured using the Nightmare Frequency Questionnaire (NFQ; [201]). Based on previous research among posttraumatic stress disorder (PTSD) patients, the NFQ appears reliable for measuring nightmare frequency [201].

##### Well-being

Well-being is measured using a single item measuring happiness [202] and the Outcome Rating Scale (ORS; [203]). The single item measures general happiness in the months prior to the assessment on a seven point Likert scale. Reliability and validity were good among undergraduates [202], and sensitivity to change was excellent in a BPD sample [184]. The ORS consists of four VAS items assessing four areas of functioning: individual (personal well-being), interpersonal (family and close relationships), social (work and/or school functioning), and overall (general sense of well-being). We slightly adapted the third dimension of the ORS by excluding friendships, because of its overlap with the second dimension (interpersonal functioning). Hafkenscheid et al. [204] reported adequate psychometric properties of the ORS is a Dutch outpatient sample.

### Statistical analyses

The statistical analyses for the (cost-)effectiveness, mechanisms of change and treatment selection studies are under development. For example, according to Cohen et al. [48], the treatment selection field is still in its developmental stage and statistical methods are constantly evolving. Recently, great efforts have been made by several authors (e.g., [205, 206]) to select the optimal prediction model by comparing different variable selection techniques. Considering the ongoing advances in methodological approaches, the statistical analyses described below should be considered as examples of appropriate analytic methods. We will determine the optimal methods at the time of the analyses. An update of the protocol will be published, including the selected statistical methods, before start of the data-analyses. The statistical analyses will be performed according to the intention-to-treat (ITT) principle (i.e., including all patients that have been randomized and received at least one treatment session). In addition to the primary analysis based on the ITT principle, a completers analysis will be conducted by excluding patients who dropped out prematurely (i.e., termination of the treatment before planned end, without patient and therapist agreeing that enough improvement has been reached to justify the termination) or deviated from the protocol (e.g., sought other psychological treatment in addition to the study treatment). No interim analyses are planned.

#### Treatment selection

A two-step approach will be applied to determine the optimal treatment for a particular patient by identifying patient characteristics that predict (differential) treatment response. First, we will examine which of the candidate predictors (see Additional file 4 for an overview) predict (differential) treatment response. Many different variable selection approaches can be used to identify which of the candidate predictors contribute to the prediction of treatment outcome, for example elastic net regularization [207], Bayesian additive regression trees [208], or a combination between different variables selection procedures [48]. Second, individual treatment recommendations are generated based on a prediction model including the variables that predict (differential) treatment response. For each patient, the most beneficial treatment will be identified by using the prediction model to estimate the predicted outcomes for both treatments including the difference in predicted outcomes.

Our primary analysis will focus on individual treatment recommendations based on change in BPD manifestations and will therefore reveal the advantage in symptom relief that may be gained if patients are allocated to their predicted optimal treatment compared to their predicted non-optimal treatment. To investigate the advantage that may be gained in other outcomes, such as functioning and cost-effectiveness, generalization analyses will be performed by testing the performance of the prediction model for these outcomes.

#### Mechanisms of change

It is hypothesized that the treatments exert a remedial effect on the frequency and severity of BPD manifestations by their impact on the BPD-treatment-specific (ST: beliefs and schema modes; DBT: emotion regulation and skills use), BPD-treatment-generic (therapeutic environment characterized by genuineness, safety, and equality), and non-specific (attachment and therapeutic alliance) mechanisms of change. Since potential mediators and outcome will be assessed multiple times, temporal patterns of change can be studied by performing mediation analysis for longitudinal data, for example multilevel autoregressive mediation analysis [209] or multilevel structural equation modeling [210]. By using advanced statistical models, the hierarchical structure of the data (repeated measures nested within patients, who in turn are nested within centers) can be taken into account and possible concurrent and temporal relations between mediators and outcome can be investigated.

#### Clinical effectiveness

Change in the outcome measures and the relative effectiveness of the two treatment conditions will be analyzed using mixed regression so that all available data are used, and taking into account the dependencies among observations nested within individuals nested within centers. Potential center effects are modeled by including a random effect which enables generalization of results outside the trial and maximizes statistical power [211]. Since group sessions in both treatments are offered in a semi-open format, patients will start with group treatment at different time points. One can imagine that patients starting treatment at the same time point are more interdependent compared to patients starting treatment at different time points. Therefore, we will take into account, if needed, the interdependency of patients. The underlying distributions of the mixed regression models will be determined based on the variable type (i.e., scale or nominal) and the distribution of residuals (e.g., normal, gamma, negative binomial).

#### Cost-effectiveness

The cost-effectiveness evaluation will be performed from a societal perspective and includes a cost-effectiveness analysis (CEA) and cost-utility analysis (CUA). The primary clinical outcome for the CEA will be the severity of the BPD manifestations and for the CUA utility scores will be derived from the quality of life instrument(s), both with a time horizon of 12 months after the end of treatment. The net benefit will be used to express cost-effectiveness. For each patient, the net benefit will be calculated by subtracting the costs incurred by the patient from the amount that the society is willing to pay for the health benefit [212]. The development of the net benefit over time and differences between the treatments will be modeled using multilevel modeling in which the hierarchical structure of the data and potential missing values are taken into account. The best fitting model to describe the development over time and the appropriate distribution of the net benefit data (e.g., gamma distribution, log-normal distribution) will be based on the data. Cost-effectiveness acceptability curves (CEACs) will be drawn showing the probability that one treatment is more cost-effective compared to the other treatment, given the observed data, for a range of willingness-to-pay values. Sensitivity analyses will be performed to address the uncertainties in methodology and assumptions and to test for the robustness of findings.

### Additional substudies

Several secondary studies will be conducted, including, but not limited to, the investigation of the heterogeneity of BPD and substance abuse among BPD patients, a qualitative study into the perspectives of patients and therapists, and psychometric evaluations. First, BPD is characterized by considerable heterogeneity [165, 213]. Over the past years, researchers have attempted to identify BPD subtypes based on different indicator variables (e.g., DSM-5 criteria, interpersonal characteristics, temperament) and different statistical strategies (e.g., exploratory factor analysis, Q-factor analysis, finite mixture modeling) [214]. The BPD subtypes that emerged differed substantially between studies. According to Hallquist and Pilkonis [214], advances in classifying BPD subtypes can be made by using a theoretical model as guidance, for example Kernberg’s theory [215]. Therefore, a substudy into the heterogeneity of BPD will be performed based on theoretically justified indicators and state-of-the-art statistical methods.

A second substudy will focus on the co-occurrence of substance abuse and BPD. Research suggests that patients with BPD and substance abuse have more severe problems, including higher rates of suicide attempts, more treatment noncompliance, and increased risk of violence, compared to BPD patients without substance abuse (e.g., [216–218]). However, few trials have assessed the effectiveness of treatments for BPD patients with substance abuse. In addition, research into the effect of BPD treatment on substance abuse is also limited [219]. Third, qualitative research will be conducted to explore the perspectives of patients and therapists in key areas, including predictors, mechanisms of change, the treatments, and the implementation of the results in clinical practice. Finally, psychometric evaluations of several Dutch questionnaires (e.g., Dialectical Behavior Therapy-Ways of Coping Checklist, Ultrashort BPD Checklist) will be performed.

## Discussion

This article described the study protocol of a multicenter RCT focusing on the (differential) treatment effectiveness of DBT and ST for patients with BPD. The primary aim of the study is to improve treatment outcome of DBT and ST for BPD patients by optimizing treatment selection through identifying patient characteristics that specify which patients will benefit most from which treatment. In addition, we aim to elucidate the change mechanisms of DBT and ST, which is crucial for improving treatments and, in turn, treatment response [51, 52, 220]. Finally, the comparative effectiveness and cost-effectiveness of DBT and ST will be compared.

This trial provides a unique opportunity to gain more insight into one of the main questions dominating the psychotherapy research agenda: “What works for whom and why?”. Although DBT and ST share some important characteristics, different interventions related to different assumed core deficits in BPD are provided [58]. As each treatment provides a different therapeutic milieu and focuses on different goals and tasks, a particular treatment may be a better fit with some patients compared to others [45]. In this study, patient characteristics of (differential) treatment response will be identified and individual treatment recommendations (DBT or ST) will be generated. In addition, for each patient, an estimate will be provided of the potential advantage in symptom relief that might be gained in case the patient was allocated to his or her indicated treatment. Moreover, the potential advantage in other outcomes, for example functioning and cost-effectiveness, will also be estimated. Knowing which treatment is most cost-effective for whom may lead to more efficient allocation of healthcare resources, which is important, as the current healthcare system is characterized by constraints in resources (e.g., people, time, budget; [221]). However, before a treatment selection procedure can be implemented in clinical practice, replication and external validation of the prediction model is needed. Subsequently, a prospective study in which the patient and clinician collaborate in selecting the optimal treatment (i.e., shared decision making; [222]), guided by treatment recommendations based on the prediction model, should be conducted to evaluate the advantage of a treatment selection procedure. By using a state-of-the-art approach, the results of the current study can serve as the starting point for future studies into personalized medicine among BPD patients, and is therefore of great importance.

In addition, this trial provides insight into the comparative (cost-)effectiveness of DBT and ST. Although the effectiveness of both treatments has been established, DBT and ST have not been directly compared. Therefore, and because outcome measures differ substantially between studies on the effectiveness of DBT or ST, hypotheses concerning the differential effectiveness can hardly be formulated. According to the “Dodo Bird effect” [223, 224], all evidence-based psychotherapies are equally effective, suggesting that DBT and ST will produce equivalent outcomes. However, a meta-analysis into the comparative effectiveness of evidence-based treatments for personality disorders demonstrated that some treatments may be more effective than others [225]. In addition, Fassbinder et al. [226] hypothesized that ST may be more effective than DBT in reducing psychiatric comorbidity and improving quality of life, while DBT may lead to a better and faster reduction in self-harming and suicidal behaviors. Moreover, although not assessed in direct comparison with ST, the meta-analysis of Storebø et al. [25] into psychological treatments for BPD indicated that DBT may be especially effective for BPD-severity, self-harm, and psychosocial functioning. They also pointed out that more research into the effects of BPD-tailored treatments, including head-to-head comparisons, is needed. By focusing on an array of outcomes, this study will extend our knowledge on the potential differential effects of DBT and ST.

This study has several strengths. First, this RCT is quite inclusive in terms of patient characteristics, and as such designed to reflect clinical practice to enhance ecological validity. Second, this trial is conducted by a research group including researchers with balanced allegiance to either ST or DBT and an independent researcher (i.e., C.J.M. Wibbelink), to prevent the potential effect of research allegiance on treatment outcomes [227]. Third, we adopt a broad view on treatment response by including outcome measures reflecting different areas of recovery (e.g., BPD symptoms, functioning, well-being). Focusing on outcomes beyond symptom reduction is in line with patients’ view on recovery [76, 77]. In addition, it follows a multi-method assessment approach, as the outcome measures include both self-report questionnaires and semi-structured interviews. Fourth, we include a large amount and broad range of patient characteristics potentially predictive of (differential) treatment response across DBT and ST. Finally, the presumed mediators and outcomes will be frequently measured on multiple time points during the treatments and mediation analyses will be performed by using state-of-the-art statistical analysis methods [228]. This allows us to establish concurrent as well as temporal relationships between the mediators and outcomes [228]. However, according to Lemmens et al. [229], understanding psychotherapeutic change may be too challenging, even in optimal research designs. Psychotherapy consists of a complex interplay of multiple mechanisms on different levels. Finding that a construct (e.g., therapeutic alliance) mediates treatment outcome does not explain *how* changes in this construct lead to changes in the outcome as it could involve several processes (e.g., cognitions, behaviors, emotions, neural systems) [63]. It is therefore highly questionable if these complex processes can be assessed by relatively simple mediational models. As such, this is one of the potential limitations of the current study.

This study has several other limitations that should be considered when evaluating the results. First, as power is conventionally set a 80% [84, 230–232], we used a minimum criterion of 80% power for the power analyses. However, this means that we accept a 20% chance of a false negative result. Second, since DBT and ST are both evidence-based treatments for BPD, differential effects in treatment outcome may be small or non-existing. To demonstrate equivalence or small effects between treatments, a very large sample size is needed. The sample size of the current study is not large enough (i.e., does not have ≥80% power) to reliably detect a small differential treatment effect. However, the comparison of treatments is not the main aim of the study. In addition, according to Luedtke et al. [233], a sample size of at least 300 patients per condition is required to have sufficient power for applying multivariable prediction models. Nonetheless, they also noticed that a smaller sample size might be justified if studies are designed to develop prediction models that can be tested in future studies. Moreover, the results of this study can contribute to building a database including trials on BPD that can be analyzed with meta-analytic techniques.

Second, this study does not include a no treatment control group, which might affect internal validity. When improvements are found in both treatments, but no significant differences between the treatments, the absence of a control group implies that it cannot be ruled out that non-specific factors such as attention or time (maturation) caused the improvements. However, including a control group receiving no treatment would clearly be unethical (e.g., patients are at risk of suicide). For similar reasons, it is not possible to standardize medication use and crisis management sessions. Any additional treatment or medication use will be monitored and included in the analyses.

Third, one of the treatment elements of DBT is out of office hours between-session (telephone) consultation by the individual therapist. The targets of telephone consultation include, among others, reducing self-harm and suicidal behavior and teaching patients how to apply learned skills in everyday life in order to encourage skills generalization [19]. In the current study, some centers provide 24/7 access to telephone consultation by the patient’s individual therapist, while the other centers provide telephone consultation within the limitations of the individual therapists, or within working hours. In case of emergency, the standard emergency procedures of each center will be followed. Although outside of office hours availability is considered to be an essential element of DBT by some authors [113, 234], the link between telephone consultation and outcome in DBT has not been evaluated [95, 235]. There is some preliminary support for the importance of telephone consultation [236]. However, studies into the effectiveness of DBT that did not apply 24-h telephone consultation by the individual therapist have found positive outcomes (e.g., [235, 237]). Van den Bosch and Sinnaeve [238] studied treatment programs of 25 DBT teams in the Netherlands. They found that only 36% of the DBT teams applied telephone consultation according to the guidelines of DBT. It can therefore be concluded that the current study is a good reflection of clinical practice, which enhances generalizability of our findings. Notwithstanding, we will monitor between-session (telephone) consultation within centers and examine potential effects.

Fourth, in this study, a component of treatment integrity (treatment adherence) will be assessed, which is, surprisingly, not standard procedure in trials investigating BPD treatments [25]. However, treatment integrity also constitutes of treatment differentiation and therapist competence [110]. Treatment adherence and treatment differentiation are closely related, in contrast to treatment adherence and therapist competence [239]. Treatment adherence represents a quantitative aspect of treatment integrity (i.e., how frequently a therapist utilizes prescribed techniques and procedures and avoids proscribed techniques and procedures), while competence represents a qualitative aspect (i.e., how well prescribed techniques and procedures are implemented) [109]. Adherence does not necessary presuppose competence; even with adequate adherence, therapists may deliver the treatment in an incompetent manner. The absence of competence ratings may threaten the validity of our results [109]. Moreover, treatment adherence will be assessed by trained master psychology students, whereas for DBT, adherence ratings by reliably trained therapists are considered the gold standard [240, 241]. However, students will receive a training from experienced therapists.

Fifth, it is a subject of some debate whether the EQ-5D is a valid instrument to measure quality of life in BPD patients, which can affect the economic evaluation [81]. According to Brazier [242], the EQ-5D might not measure what matters to patients with psychiatric disorders. In addition, in a cost-effectiveness study among BPD patients, van Asselt et al. [79] found contradictory results on the incremental risk ratios when recovery was based on the EQ-5D compared to the BPDSI-IV. In contrast, adequate responsiveness of the EQ-5D has been found in a BPD sample [243]. In addition, Soeteman et al. [244] concluded that the EQ-5D is sensitive to changes in the health status of patients with cluster B personality disorders. In addition to the EQ-5D, quality of life will be assessed by a recently developed instrument specially developed for patients with mental health problems (MHQoL-7D; [197]). The validation of this instrument is currently in progress, but preliminary results are promising [197]. Another point for consideration is that conclusions with regard to the most cost-effective treatment choice can be affected by the amount the society is willing to pay for an additional unit of effectiveness (i.e., willingness-to-pay threshold). Soeteman et al. [244] concluded that outpatient psychotherapy for cluster B personality disorder patients is the optimal treatment choice in case society is not willing to pay more than €12.274; otherwise, day hospital psychotherapy was the optimal treatment choice. To date, there is no consensus about reasonable willingness-to-pay thresholds, although guidelines have been proposed by the Dutch healthcare authority [245]. We will therefore calculate the probability of each treatment being cost-effective for different willingness-to-pay values. As a result, the optimal treatment choice can be different for different willingness-to-pay values.

Finally, this study is conducted during the COVID-19 pandemic. The COVID-19 pandemic has significant disrupted effects on society and is related to increased burden of mental health among individuals with mental disorders [246, 247]. Moreover, some authors suggest that patients with severe psychopathology, including BPD, may be especially at risk for symptom deterioration [247, 248]. In addition, some patients will temporarily receive treatment via videoconferencing in case face-to-face treatment is restricted in mental healthcare centers. Research on the effectiveness of online individual psychotherapy has found positive effects for several mental health disorders, including PTSD [249], anxiety disorders [250], and depression [251]. However, research on the effectiveness of online group psychotherapy is scarce [95, 252]. Consequently, we will control for a potential effect of the COVID-19 pandemic in the analyses.

## Conclusion

Specialized evidence-based treatments have been developed and evaluated for BPD, including DBT and ST. However, BPD patients vary widely in their response to treatment, and poor response to one treatment does not imply poor response to another treatment. The selection of the optimal treatment for a particular patient is a daily task of the clinician, but very scant evidence is available to guide these decisions. This study will extend our knowledge on one of the main issues in psychotherapy research; understanding for whom a treatment works and how. As such, this study helps pave the way for an evidence-based personalized medicine for patients with BPD.

## Trial status

Recruitment has started in January 2019 and is still ongoing. The estimated completion date of the recruitment is September 2021. Protocol version 07 is currently active.

## Supplementary Information

**Additional file 1.** SPIRIT 2013 checklist.

**Additional file 2.** Informed consent form (Appendix A) and additional informed consent form videoconferencing (Appendix B).

**Additional file 3.** Overview of syndrome disorders assessed with the SCID-5-S.

**Additional file 4.** Candidate predictors based on clinicians’ appraisals (Table 1) and candidate predictors based on the literature and suggestions of a patient representative of the Borderline Foundation of the Netherlands (Table 2).

## Data Availability

Not applicable.

## Abbreviations

AQ-10: Autism Spectrum Quotient-10 items
BEAQ: Brief Experiential Avoidance Questionnaire
BPD: Borderline Personality Disorder
BPDSI-5: Borderline Personality Severity Index, fifth edition
BSI: Brief Symptom Inventory
CAT-PD-SF: Computerized Adaptive Test of Personality Disorder-Static Form
CEA: Cost-effectiveness analysis
CEACs: Cost-effectiveness acceptability curves
CUA: Cost-utility analysis
DART: Dutch Adult Reading Test
DBT: Dialectical Behavior Therapy
DBT-WCCL: Dialectical Behavior Therapy-Ways of Coping Checklist
DERS-18: Difficulties in Emotion Regulation Scale 18
DERS-SF: Difficulties in Emotion Regulation Scale Short Form
DSM: Diagnostic and Statistical Manual of Mental Disorders
ECR-R: Experience in Close Relationships-Revised
ECR-RS: Experiences in Close Relationships-Relationship Structures questionnaire
EQ-5D-5L: 5-level EuroQol 5D version
F-MPS-Brief: Frost Multidimensional Perfectionism Scale-Brief
GPM: General Psychiatric Management
IAPT: Improving Access to Psychological Therapies
IE: Locus of Control scale
ISI: Insomnia Severity Index
ITT: Intention-to-treat
LPFS-BF 2.0: Level of Personality Functioning Scale-Brief Form 2.0
MEC-AMC: Medical Ethics Committee of the Academic Medical Center
MHQoL-7D: Mental Health Quality of Life seven-dimensional Questionnaire
MMPI-2-RF: Minnesota Multiphasic Personality Inventory-2 Restructured Form
MSPSS: Multidimensional Scale of Perceived Social Support
NFQ: Nightmare Frequency Questionnaire
ORS: Outcome Rating Scale
PMH-scale: Positive Mental Health scale
PTSD: Posttraumatic Stress Disorder
QALYs: Quality Adjusted Life Years
RCT: Randomized Clinical Trial
RFQ-8: 8-item Reflective Functioning Questionnaire
SCID-5: Structured Clinical Interview for DSM-5
SCID-5-CV: Structured Clinical Interview for DSM-5 Disorders Clinician Version
SCID-5-PD: Structural Clinical Interview for DSM-5 Personality Disorders
SCID-5-S: Structural Clinical Interview for DSM-5 Syndrome Disorders
SIPP-118: Severity Indices of Personality Problems
SCID-5-SPQ: Structured Clinical Interview for DSM-5 Screening Personality Questionnaire
SCID-5-SV: Structured Clinical Interview for DSM-5 Syndroomstoornissen Vragenlijst
SMI: Schema Mode Inventory
SPIRIT: Standard Protocol Items: Recommendations for Interventional Trials
SRIS: Self-Reflection and Insight Scale
ST: Schema Therapy
TEC: Traumatic Experience Checklist
TFP: Transference Focused Psychotherapy
TIPI: Ten-Item Personality Inventory
TMS-F: Treatment Motivation Scales for Forensic Outpatient Treatment
TVIC: Gordon Test of Visual Imagery Control
URICA: University of Rhode Island Change Assessment
VAS: Visual Analogue Scale
WAI-S: Working Alliance Inventory-Short
WHODAS 2.0: World Health Organization Disability Assessment Schedule 2.0
