# Supplementary material for: Towards optimal treatment selection for borderline personality disorder patients (BOOTS): a study protocol for a multicenter randomized clinical trial comparing schema therapy and dialectical behavior therapy
Source: BMC Psychiatry. 2022 Feb 5;22:89. doi: 10.1186/s12888-021-03670-9 (PMC8817780; doi:10.1186/s12888-021-03670-9)
Supplement: Supplementary file 2 — Additional file 2. Informed consent form (Appendix A) and additional informed consent form videoconferencing (Appendix B). [file 12888_2021_3670_MOESM2_ESM.docx]

**Additional file 2**

Informed Consent Forms

**Appendix A: Informed consent form**

1. I have read the information sheet. I was able to ask questions. My questions have been answered well enough. I had enough time to decide if I wanted to take part.
2. I know that taking part is voluntary. I understand I can withdraw from the study at any time. I do not have to give a reason for withdrawing.
3. I give the consent to inform my doctor that I am taking part in this study.
4. I know that some people will be able to see all of my data to review the study. These people are mentioned in the information sheet.
5. I give consent to collect and use my (anonymized) data as described in the information sheet.
6. I give consent to store my (anonymized) data for 15 years after termination of the study and the recordings of the treatment sessions for a maximum of five years after termination of the study.
7. I agree to participate in the study.

Name:

Date of birth:

Signature: Date:

1. I give/give not (*circle appropriate*) consent to ask me after this study if I want to participate in a follow-up study.

Signature: Date:

I declare that I have fully informed this subject about the study mentioned.

If any information becomes known during the study that could influence the subject's consent, I will let this subject know in good time.

Name:

Position:

Signature: Date:

**Appendix B: Informed consent form videoconferencing**

1. I have read and understand the information sheet.
2. I was able to ask questions. My questions have been answered well enough.
3. I had enough time to decide if I agree with the temporary changes.
4. I know that giving my consent for the temporary changes is voluntary. I understand I can withdraw my consent at any time.
5. I agree with 1) receiving the treatment via videoconferencing and 2) conducting the assessments via videoconferencing/phone (interviews) and online (questionnaires).

Name:

Date of birth:

Signature: Date:

I declare that I have fully informed this subject.

Name:

Position:

Signature: Date:
