# Supplementary material for: Towards optimal treatment selection for borderline personality disorder patients (BOOTS): a study protocol for a multicenter randomized clinical trial comparing schema therapy and dialectical behavior therapy
Source: BMC Psychiatry. 2022 Feb 5;22:89. doi: 10.1186/s12888-021-03670-9 (PMC8817780; doi:10.1186/s12888-021-03670-9)
Supplement: Supplementary file 3 — Additional file 3. Overview of syndrome disorders assessed with the SCID-5-S. [file 12888_2021_3670_MOESM3_ESM.docx]

**Additional file 3**

Overview of Syndrome Disorders Assessed with the SCID-5-S

| **Module** | **Content** | **Time Period** |
| --- | --- | --- |
| *Module A: Mood Episodes and Persistent Depressive Disorder* | Major Depressive Episode | Current (past month) and past |
|  | Manic Episode | Current (past month) and past |
|  | Hypomanic Episode | Current (past month) and past |
|  | Persistent Depressive Disorder | Current (past 2 years) |
| *Module B: Psychotic and Associated Symptoms* | Delusions | Lifetime |
|  | Hallucinations | Lifetime |
|  | Disorganized Speech and Behaviour | Lifetime |
|  | Catatonic Behaviour | Lifetime |
|  | Negative Symptoms | Lifetime |
| *Module C: Differential Diagnosis of Psychotic Disorders* | Schizophrenia | Current (past month) and past |
|  | Schizophreniform Disorder | Current (past month) and past |
|  | Schizoaffective Disorder | Current (past month) and past |
|  | Delusional Disorder | Current (past month) and past |
|  | Brief Psychotic Disorder | Current (past month) and past |
|  | Other Specified Psychotic Disorder | Current (past month) and past (remission) |
|  | Psychotic Disorder Due to AMC | Lifetime |
|  | Substance/Medication‐Induced Psychotic Disorder | Lifetime |
| *Module D: Differential Diagnosis of Mood Disorders* | Bipolar I Disorder | Current (past month) and past (remission) |
|  | Bipolar II Disorder | Current (past month) and past (remission) |
|  | Other Specified/Unspecified Bipolar Disorder | Current (past month) and past (remission) |
|  | Bipolar Disorder Due to AMC | Lifetime |
|  | Substance/Medication-Induced Bipolar Disorder | Lifetime |
|  | Major Depressive Disorder | Current (past month) and past (remission) |
|  | Other Specified/Unspecified Depressive Disorder | Current (past month) and past (remission) |
|  | Depressive Disorder Due to AMC | Lifetime |
|  | Substance/Medication-Induced Depressive Disorder | Lifetime |
| *Module E: Substance Use Disorders* | Alcohol Use Disorder | Current (past 12 months) |
|  | Sedative, Hypnotic, or Anxiolytic Use Disorder | Current (past 12 months) |
|  | Cannabis Use Disorder | Current (past 12 months) |
|  | Stimulant Use Disorder | Current (past 12 months) |
|  | Opioid Use Disorder | Current (past 12 months) |
|  | Phencyclidine Use Disorder | Current (past 12 months) |
|  | Other Hallucinogen Use Disorder | Current (past 12 months) |
|  | Inhalant Use Disorder | Current (past 12 months) |
|  | Sedative/Hypnotic/Anxiolytic Use Disorder | Current (past 12 months) |
|  | Other or Unknown Substance Use Disorder | Current (past 12 months) |
|  | Gambling Disorder | Current (past 12 months) |
| *Module F: Anxiety Disorders* | Panic Disorder | Current (past month) and past |
|  | Agoraphobia | Current (past 6 months) |
|  | Social Anxiety Disorder | Current (past 6 months) |
|  | Generalized Anxiety Disorder | Current (past 6 months) |
|  | Anxiety Disorder Due to AMC | Lifetime |
|  | Substance/Medication‐Induced Anxiety Disorder | Lifetime |
|  | Specific Phobia | Current (past 6 months) |

| **Module** | **Content** | **Time Period** |
| --- | --- | --- |
| *Module G: Obsessive‐*  *Compulsive and Related Disorders* | Obsessive‐Compulsive Disorder | Current (past month) |
|  | OC and Related Disorder Due to AMC | Lifetime |
|  | Substance/Medication‐Induced Obsessive‐Compulsive and Related Disorder | Lifetime |
|  | Body Dysmorphic Disorder | Current (past month) |
| *Module H: Posttraumatic Stress Disorder* | Posttraumatic Stress Disorder | Current (past month) and past |
| *Module I: Externalising Disorders* | Attention‐Deficit/Hyperactivity Disorder | Current (past 6 months) |
|  | Intermittent Explosive Disorder | Current (past 12 months) |
| *Module J: Feeding and Eating Disorders* | Anorexia Nervosa | Current (past 3 months) and past |
|  | Bulimia Nervosa | Current (past 3 months) |
|  | Binge‐Eating Disorder | Current (past 3 months) |
|  | Avoidant/Restrictive Food Intake Disorder | Current (past month) |
|  | Other Specified Feeding or Eating Disorder | Current |
| *Module K: Somatic Symptom and Related Disorders* | Somatic Symptom Disorder | Current (past 6 months) |
|  | Illness Anxiety Disorder | Current (past 6 months) |
| *Module L: Sleep Disorders* | Insomnia Disorder | Current (past 3 months) |
|  | Hypersomnolence Disorder | Current (past 3 months) |
|  | Nightmare disorder | Current (past month) |
| *Module M: Screening for other disorders* | Separation Anxiety Disorder | n/a |
|  | Hoarding Disorder | n/a |
|  | Trichotillomania | n/a |
|  | Excoriation Disorder | n/a |
| *Module N: Adjustment disorder* | Adjustment disorder | Current (past 6 months) |

*Note*. n/a = not available
