# Supplementary material for: Towards optimal treatment selection for borderline personality disorder patients (BOOTS): a study protocol for a multicenter randomized clinical trial comparing schema therapy and dialectical behavior therapy
Source: BMC Psychiatry. 2022 Feb 5;22:89. doi: 10.1186/s12888-021-03670-9 (PMC8817780; doi:10.1186/s12888-021-03670-9)
Supplement: Supplementary file 4 — Additional file 4. Candidate predictors based on clinicians’ appraisals (Table 1) and candidate predictors based on the literature and suggestions of a patient representative of the Borderline Foundation of the Netherlands (Table 2). [file 12888_2021_3670_MOESM4_ESM.docx]

**Additional file 4**

Candidate Predictors

Table 1

*Candidate Predictors Based on* *Clinicians’ Appraisals*

| Domains and (sub)categories | | | |
| --- | --- | --- | --- |
| Personality and psychopathology | | | |
| Comorbidity | | | |
|  | Syndrome disorders | | |
|  |  | Primary syndrome disorder | |
|  |  | Substance use disorder | |
|  |  | Depressive disorder | |
|  |  | Anxiety disorder | |
|  |  | Eating disorder | |
|  |  |  | Anorexia nervosa |
|  |  |  | Bulimia nervosa |
|  |  | Posttraumatic stress disorder | |
|  |  |  | Re-experiencing symptoms |
|  |  | Developmental disorder | |
|  |  |  | Autism |
|  | Personality disorders | | |
|  |  | Cluster A | |
|  |  |  | Paranoid personality disorder |
|  |  |  | Schizoid personality disorder |
|  |  |  | Schizotypal personality disorder |
|  |  | Cluster B | |
|  |  |  | Antisocial personality disorder |
|  |  |  | Narcissistic personality disorder |
|  |  | Cluster C | |
|  |  |  | Avoidant personality disorder |
|  |  |  | Obsessive-compulsive personality disorder |
|  |  |  | Dependent personality disorder |
|  | Somatic symptom disorder | | |
|  | Physical diseases | | |
| Borderline personality organization (low level vs. high level) | | | |
| Duration of BPD manifestations | | | |
| Internalizing BPD manifestations | | | |
| Feelings of emptiness | | | |
| Separation anxiety | | | |
| Identity disturbance | | | |
| (Self-)destructive behavior | | | |
| Self-harm | | | |
| Suicidal behavior | | | |
| Acting out/impulse control problems | | | |
|  | | | |
|  |  | | |
|  |  | | |

Table 1 (continued)

| Domains and (sub)categories |
| --- |
| Personality traits |
| Antisocial |
| Narcissistic |
| Avoidant |
| Obsessive-compulsive |
| Psychotic symptoms |
| Dissociation |
| Flexibility |
| Pretend mode / detached protector mode |
| Self-criticism |
| Perfectionism |
| Worrying / ruminate |
| Distrust |
| Emotion regulation problems |
| Hostility |
| Crisis susceptibility |
| Frustration tolerance |
| Attachment |
| Locus of control |
| Alexithymia |
| Experiential avoidance / emotional avoidance |
| Mental imagery capacity |
| Untrustworthy |
| Curious / eager to learn |
| Self-discipline |
| Level of Personality Functioning |
|  |
| Capacity and skills |
| Cognitive abilities |
| Mentalizing capacity |
| Insight |
| Link childhood history to present problems (unable / unwilling) |
| Coping skills |
| Social skills |
| Skills to deal with problems and stresses |
|  |
| Context |
| Psychosocial and environmental problems |
| Problems related to the social environment |
| Economic problems |
| Housing problems |
| Occupational and/or educational problems |
| Living in a destructive environment |
| Child living at home |

Table 1 (continued)

| Domains and (sub)categories |
| --- |
| History |
| Trauma |
| Adoption background |
|  |
| Demographics |
| Age |
|  |
| Treatment |
| Motivation / willingness to change |
| Treatment history |
| Willingness and/or ability to engage in a therapeutic relationship |
| Commitment to treatment |
| Confidence in treatment |
| Preference for treatment |
| Request for help |
|  |
| Other |
| Hope |
| Degree of suffering |
| Separation problems |
| Disruption of day-night rhythm |
| Positive mental health |
| Self-stigma |

*Note*. BPD = borderline personality disorder.

Table 2

*Candidate Predictors Based on the Literature and Suggestions of a Patient Representative of the Borderline Foundation of the Netherlands*

| Domains and (sub)categories | |
| --- | --- |
| Personality and psychopathology | |
| Bipolar disorder | |
| Schizophrenia | |
| Severity of BPD manifestations | |
| Personality traits | |
|  | Paranoid |
|  | Dependent |
| Healthy adult mode | |
| Self-criticism | |
| Querulous person | |
| Hypochondriac | |
|  | |
| Context | |
| Daytime activities | |
|  | |
| History | |
| Absence of an attachment figure | |
| Divorce of parents | |
| Family with history of mental illness | |
| Bullied heavily | |
| Intimate relationship for at least six months | |
|  | |
| Demographics | |
| Ethnicity | |
| Gender | |
| Level of education | |
| Socioeconomic status | |
| Pregnancy | |
|  | |
| Other | |
| Dishonesty | |
| Medication use | |
| Unwilling to do homework | |
| Pet | |

*Note*. BPD = borderline personality disorder.
